# Supplementary material for: Epigenetic regulation of beta-endorphin synthesis in hypothalamic arcuate nucleus neurons modulates neuropathic pain in a rodent pain model
Source: Nat Commun. 2023 Nov 9;14:7234. doi: 10.1038/s41467-023-43022-7 (PMC10636187; doi:10.1038/s41467-023-43022-7)
Supplement: Supplementary file 1 — Supplementary Information [file 41467_2023_43022_MOESM1_ESM.pdf]

Supporting Information

Epigenetic regulation of beta-endorphin synthesis in hypothalamic arcuate nucleus neurons modulates neuropathic pain in a rodent pain model

Yu Tao [1,#], Yuan Zhang [2,3,#], Xiaohong Jin [4,#], Nan Hua [1], Hong Liu [4], Renfei Qi [1], Zitong Huang [1], Yufang Sun [1,3], Dongsheng Jiang [5], Terrance P. Snutch [6], Xinghong Jiang [1,3], Jin Tao [1,3,*]

[1] Department of Physiology and Neurobiology & Centre for Ion Channelopathy, Suzhou Medical College of Soochow University, Suzhou 215123, P.R. China;

[2] Department of Geriatrics & Clinical Research Center of Neurological Disease, The Second Affiliated Hospital of Soochow University, Suzhou 215004, P.R. China;

[3] Jiangsu Key Laboratory of Neuropsychiatric Diseases, Soochow University, Suzhou 215123, P.R. China;

[4] Department of Pain Medicine, The First Affiliated Hospital of Soochow University, Suzhou 215006, P.R. China;

[5] Institute of Regenerative Biology and Medicine, Helmholtz Zentrum München, Munich 81377, Germany;

[6] Michael Smith Laboratories and Djavad Mowafaghian Centre for Brain Health, University of British Columbia, Vancouver, BC, V6T 1Z4, Canada;

[#] These authors contribute to this work equally.

***To whom correspondence should be addressed:** Dr. Jin Tao, Department of Physiology and Neurobiology & Centre for Ion Channelopathy, Suzhou Medical College of Soochow University, 199 Ren-Ai Road, Suzhou 215123, P.R. China. E-mail: taoj@suda.edu.cn

This PDF file includes: SI Figures S1 to S25

SI Tables S1 and S4

SI Figures S1 to S25

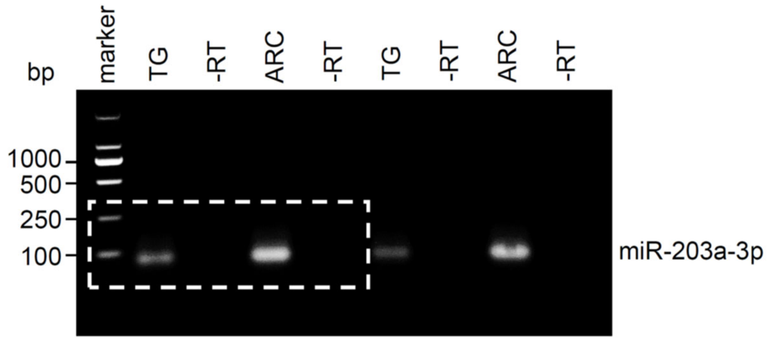

Fig. S1: RT-PCR analysis of miR-203a-3p in the ARC and TGs of naïve rats. Shown is the expanded image of RT-PCR for miR-203a-3p presented in Fig. 1h.

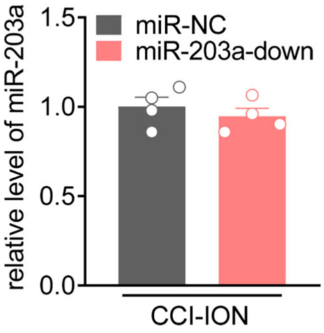

Fig. S2: qPCR analysis of miR-203a-3p in the ARC of CCI-ION rats after administration of miR-NC or miR-203a-down.

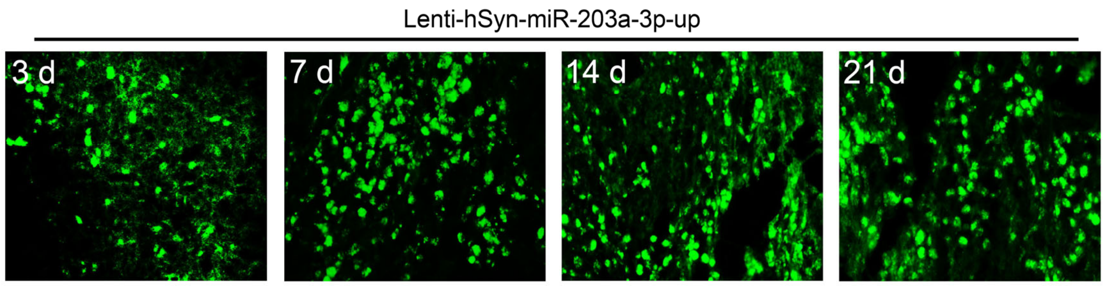

Fig. S3: Validation of lentiviral delivery in the ARC of naïve rats. The expression of lenti-hSyn-miR-203a-3p-up (miR-203a-up) carrying the construct encoding eGFP was observed in ARC neurons at 3 days after intra-ARC injection, and was maintained on day 21.

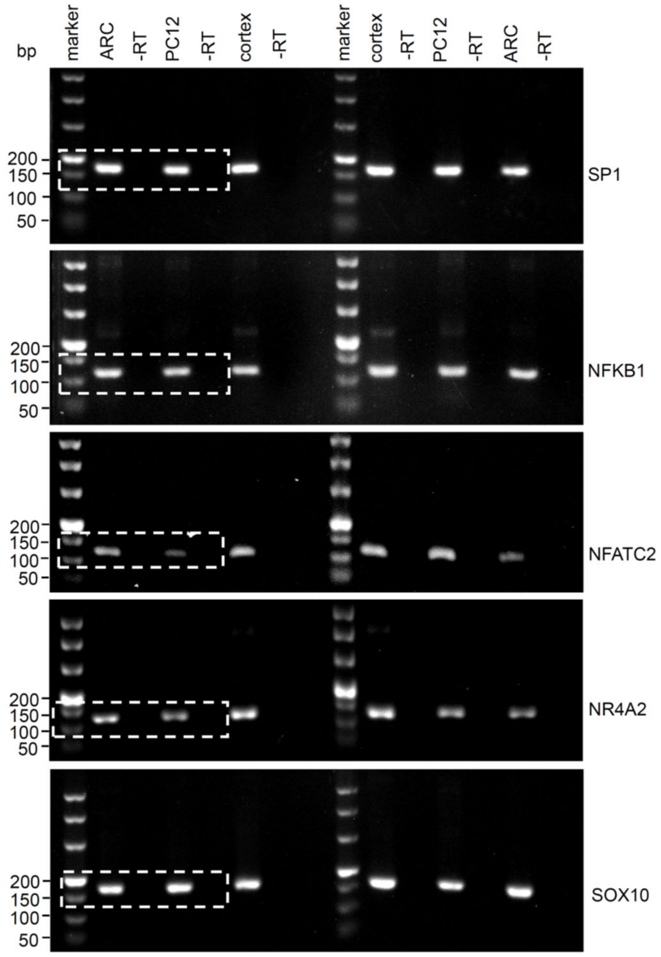

Fig. S4: RT-PCR analysis of SP1, NFKB1, NFATC2, NR4A2, and SOX10 in rat ARCs, rat cortex and PC12 cells. Shown are the expanded images of RT-PCR presented in Fig. 3d.

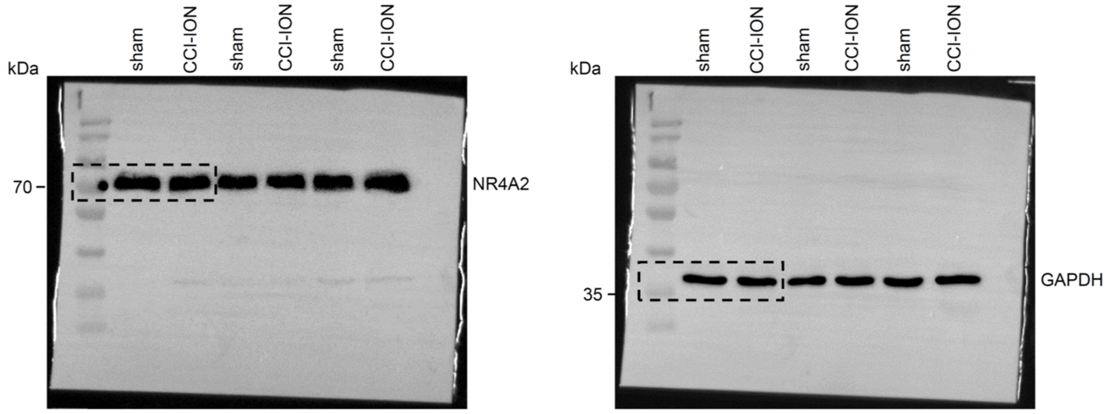

Fig. S5: Protein expression of NR4A2 in rat ARCs after CCI-ION. Shown are the expanded images of Western blots for NR4A2 against a loading control, GAPDH, presented in Fig. 3g. Blots are representative of at least three experiments.

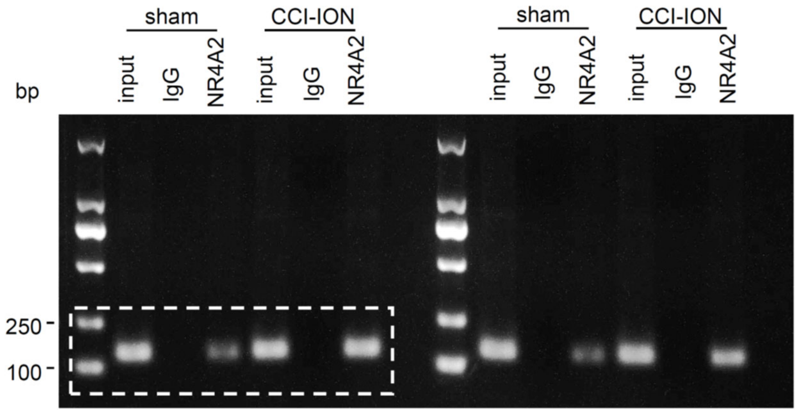

Fig. S6: Blots of ChIP-qPCR analysis show the binding activity of NR4A2 in the miR-203a-3p gene promoter in the rat ARC after CCI-ION or sham surgery. Shown is the expanded image presented in Fig. 3i.

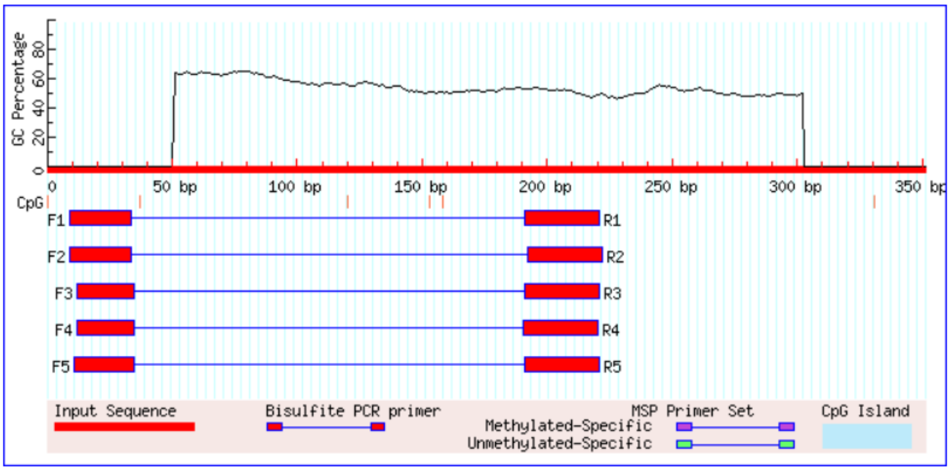

Sequence Name:
Sequence Length: 351

CpG island prediction results
(Criteria used: Island size > 100, GC Percent > 50.0, Obs/Exp > 0.6)
No CpG islands were found in your sequence

Fig. S7: Bioinformatics prediction by MethPrimer suggested that no CpG islands were found within the ΔF region of the *miR-203a-3p* gene promoter

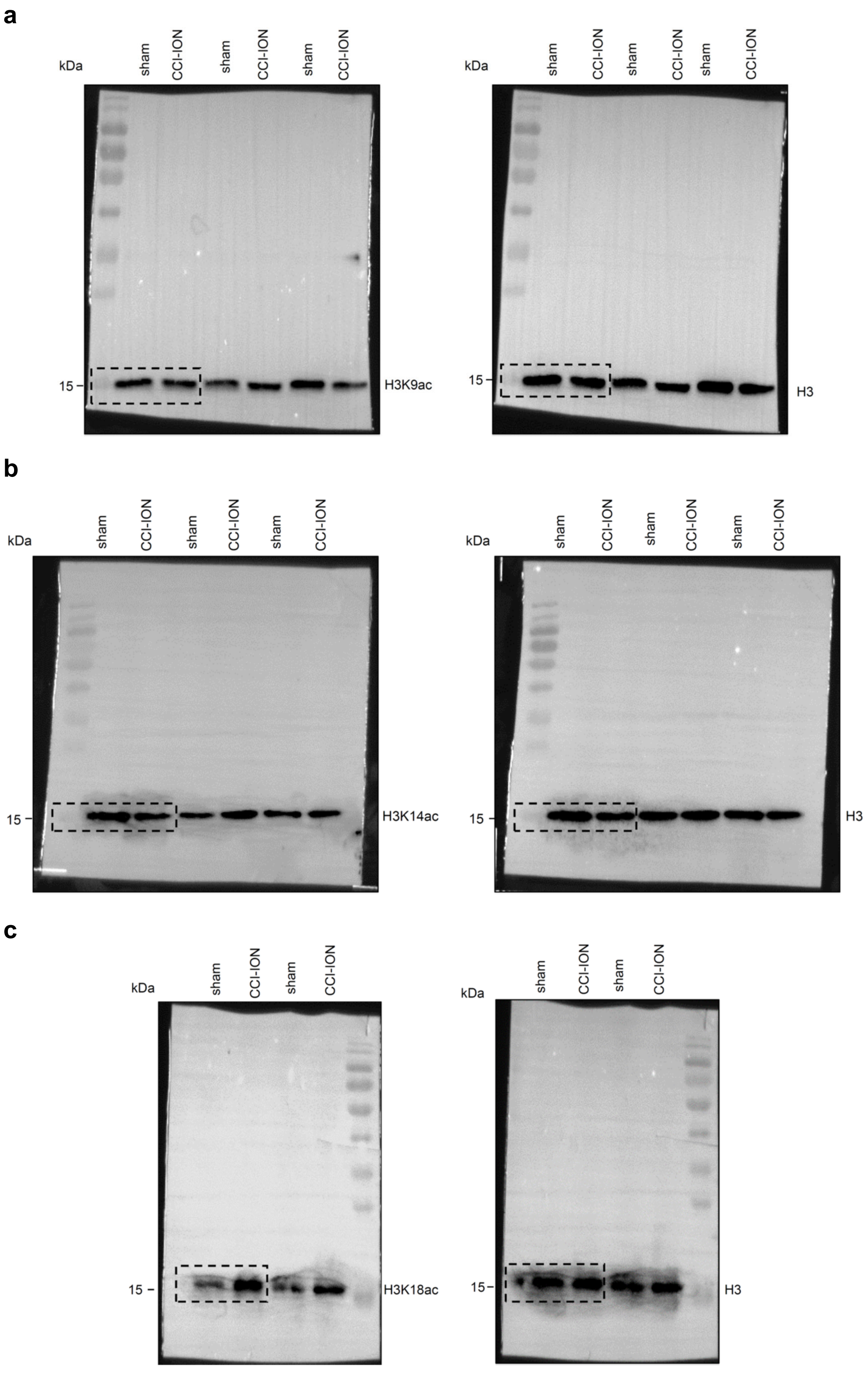
a
kDa
sham
CCI-ION
sham
CCI-ION
sham
CCI-ION
15
H3K9ac
kDa
sham
CCI-ION
sham
CCI-ION
sham
CCI-ION
15
H3
b
kDa
sham
CCI-ION
sham
CCI-ION
sham
CCI-ION
15
H3K14ac
kDa
sham
CCI-ION
sham
CCI-ION
sham
CCI-ION
15
H3
c
kDa
sham
CCI-ION
sham
CCI-ION
15
H3K18ac
kDa
sham
CCI-ION
sham
CCI-ION
15
H3

d

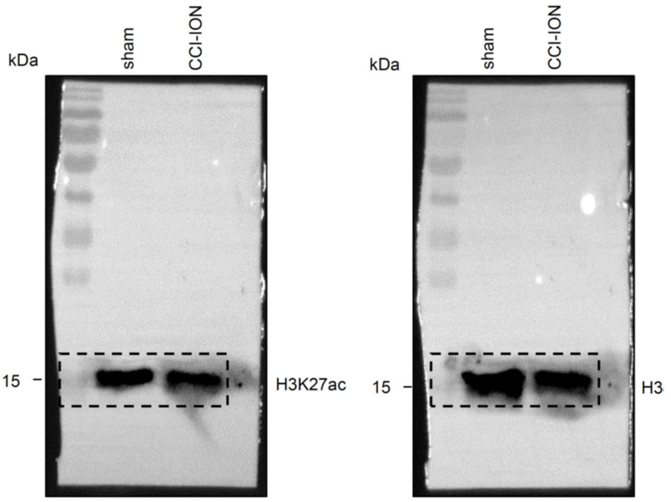

e

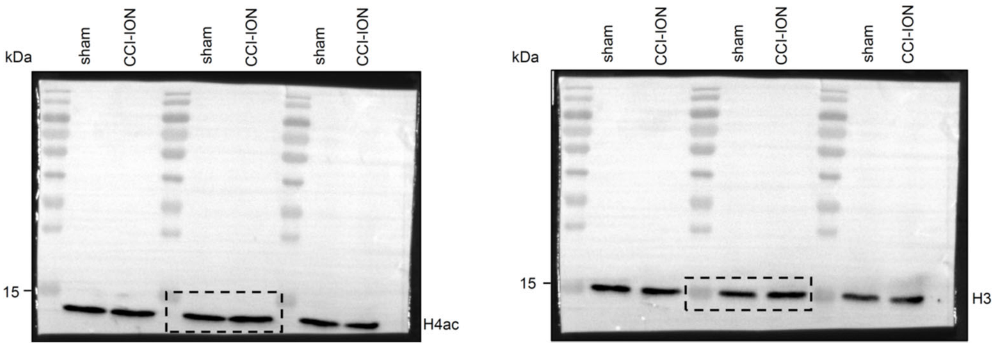

Fig. S8: Immunoblots showing the protein expression levels of H3K9ac (*a*), H3K14ac (*b*), H3K18ac (*c*), H3K27ac (*d*), and H4ac (*e*) in rat ARCs after CCI-ION or sham-operation. Shown are the expanded images of Western blots presented in Fig. 4a. Blots are representative of at least three experiments.

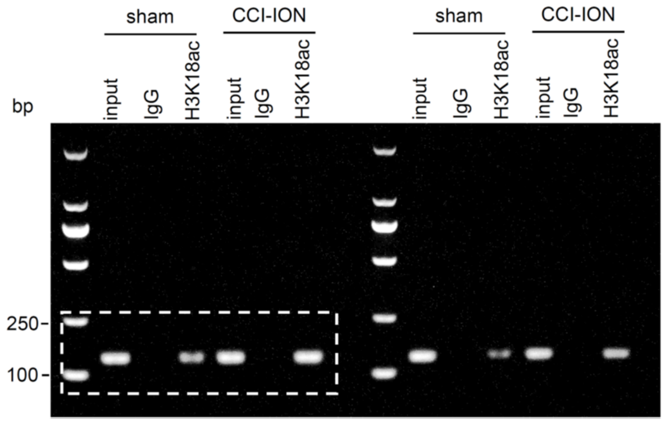

Fig. S9: Blots of ChIP-qPCR analysis show that the binding activity of H3K18ac. Shown is the expanded image presented in Fig. 4c.

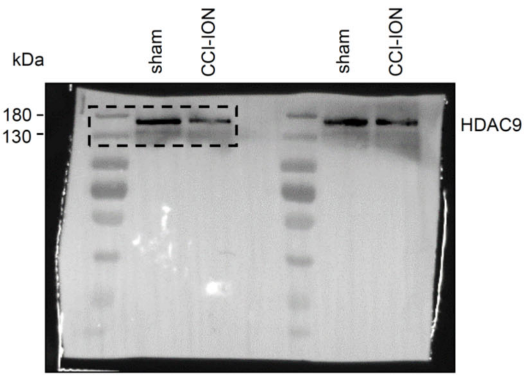

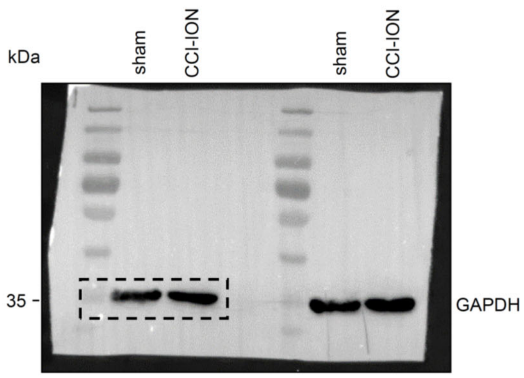

Fig. S10: Protein expression of HDAC9 in rat ARCs after peripheral nerve injury. Shown are the expanded images of Western blots for HDAC9 against a loading control, GAPDH, presented in Fig. 4e. Blots are representative of three experiments.

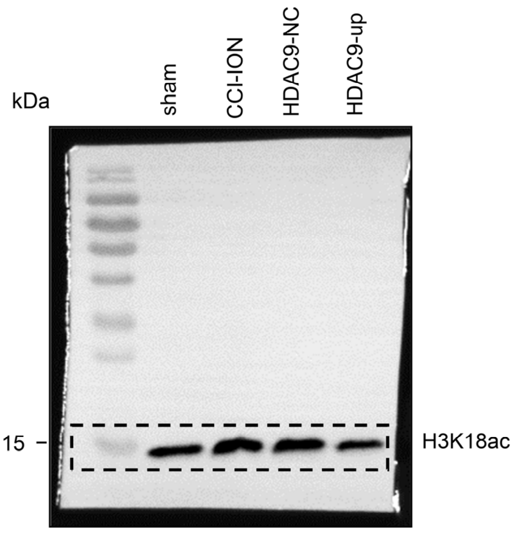

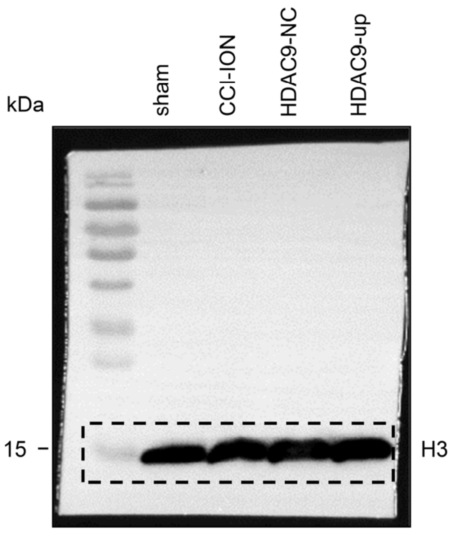

Fig. S11: Bilateral intra-ARC injection of HDAC9-up attenuated the increased expression of H3K18ac in the ARCs on day 14 post-CCI-ION. Shown are the expanded images presented in Fig. 4g. Blots are representative of at least three experiments.

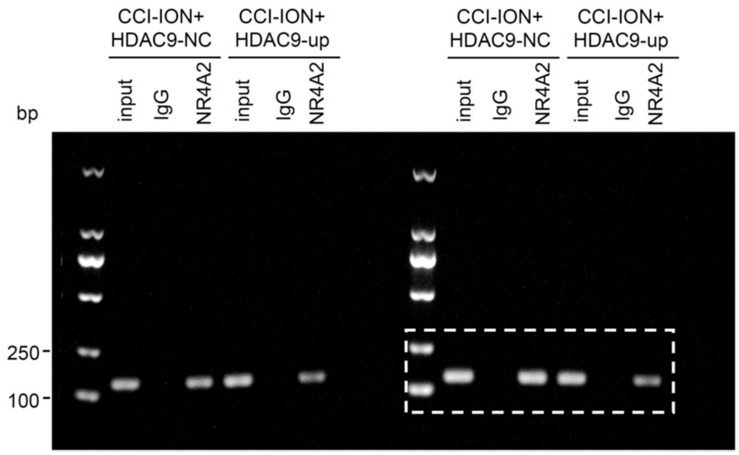

Fig. S12: Administration of HDAC9-up decreased the binding of NR4A2 to the promoter region of the m*iR-203a-3p* gene in the ARCs on day 14 post-CCI-ION. Shown is the expanded image presented in Fig. 4i.

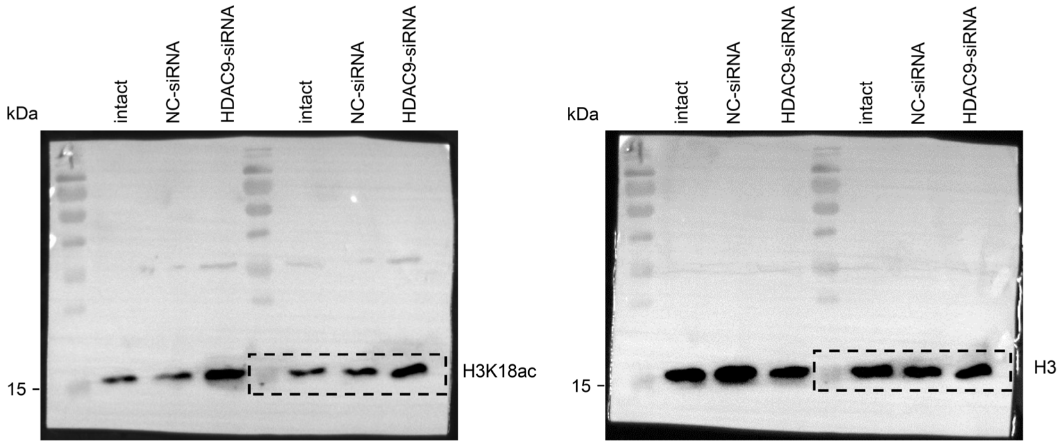

Fig. S13: Intra-ARC injection of HDAC9-siRNA attenuated the expression of H3K18ac in the ARC of intact rats. Shown are the expanded images presented in Fig. 4k. Blots are representative of at least three experiments.

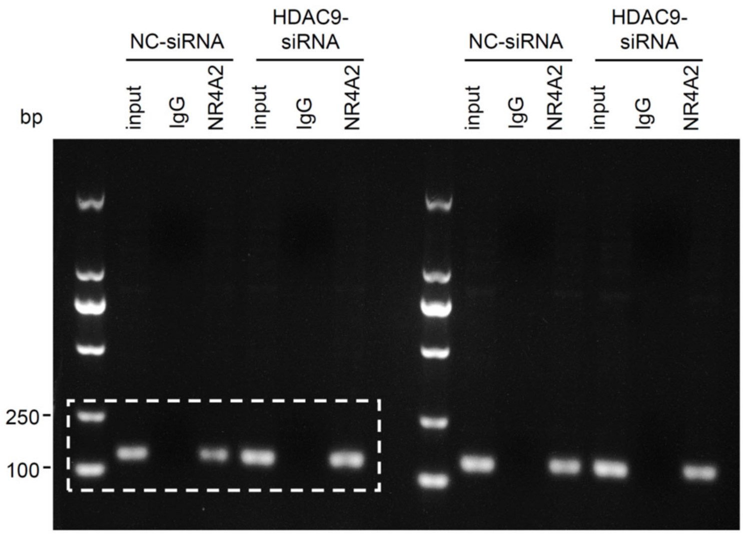

Fig. S14: Administration of HDAC9-siRNA increased the binding of NR4A2 to the promoter region of the m*iR-203a-3p* gene in the ARCs of intact rats. Shown is the expanded image presented in Fig. 4m.

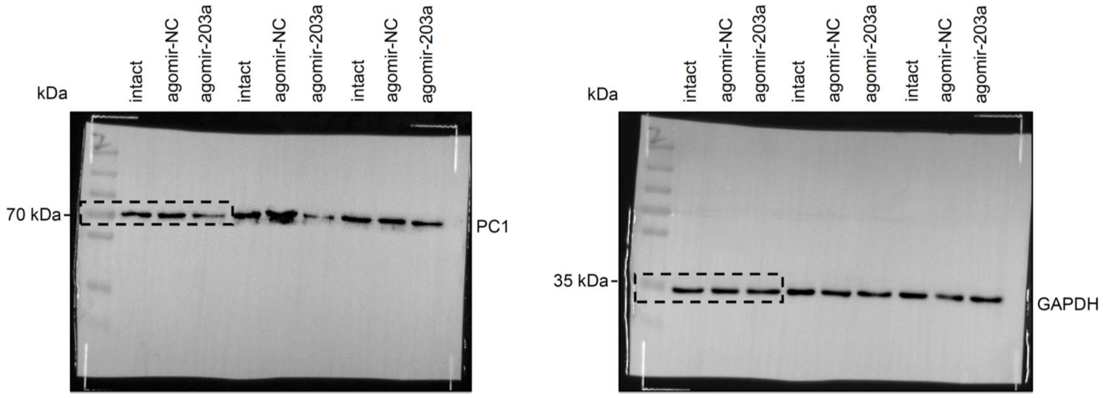

Fig. S15: Intra-ARC injection of agomir-203a decreased the protein expression of PC1 in intact rats. Shown are the expanded images of Western blots for PC1 against a loading control, GAPDH, presented in Fig. 5h. Blots are representative of at least three experiments.

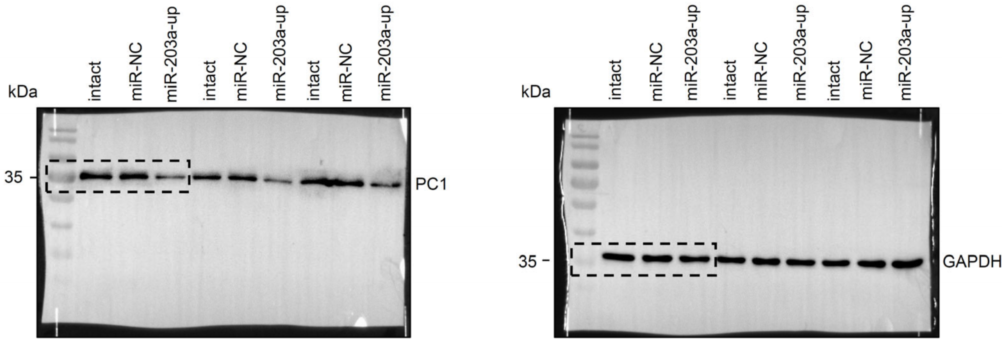

Fig. S16: Intra-ARC injection of miR-203a-up decreased the protein expression of PC1 in intact rats. Shown are the expanded images of Western blots for PC1 against a loading control, GAPDH, presented in Fig. 5i. Blots are representative of at least three experiments.

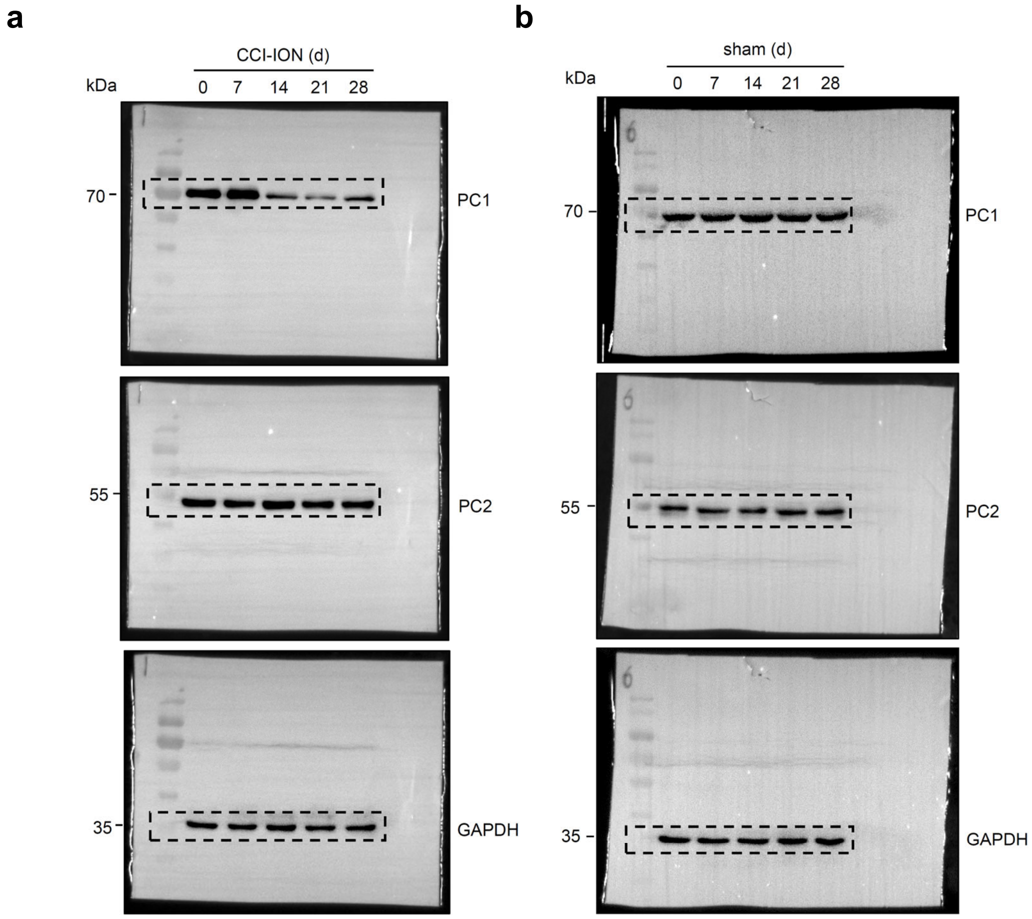

Fig. S17: Protein expression of PC1 and PC2 in the ARCs on days 0, 7, 14, 21, and 28 after CCI-ION (*a*) or sham surgery (*b*). Shown are the expanded images of Western blots for PC1 and PC2 against a loading control, GAPDH, respectively presented in Figs. 6a and b. Blots are representative of at least three experiments.

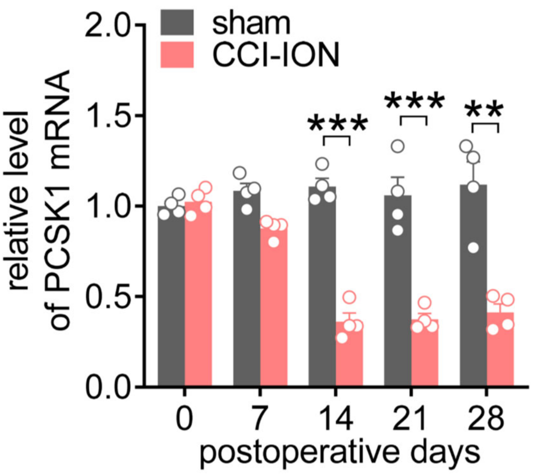

Fig. S18: Time course of PCSK1 mRNA expression by qPCR analysis in the ARC following sham or CCI-ION operation. $**p < 0.01$, $***p < 0.001$ versus sham at the corresponding time point, one-way ANOVA followed by Bonferroni's test. $n = 8$ rats per time point per group.

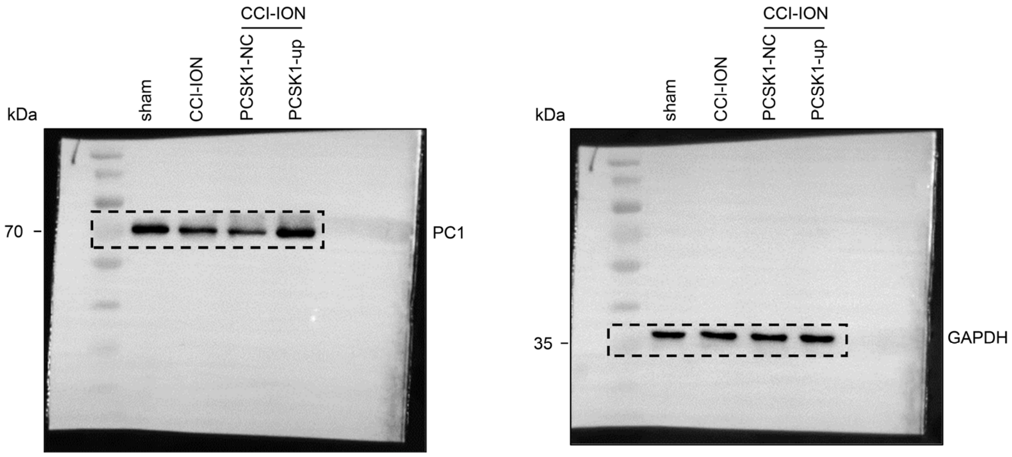

Fig. S19: The decreased expression level of PC1 induced by CCI-ION was reversed by intra-ARC injection of PCSK1-up. Shown are the expanded images of Western blots for PC1 against a loading control, GAPDH, presented in Fig. 6e. Blots are representative of at least three experiments.

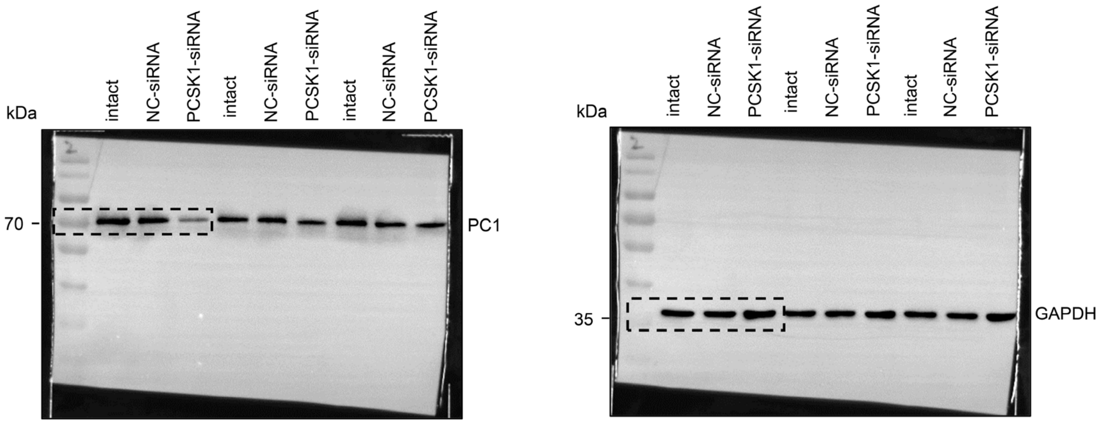

Fig. S20: Bilateral intra-ARC injection of PCSK1-siRNA attenuated the expression of PC1 in the ARCs of intact rats. Shown are the expanded images respectively presented in Fig. 6j. Blots are representative of at least three experiments.

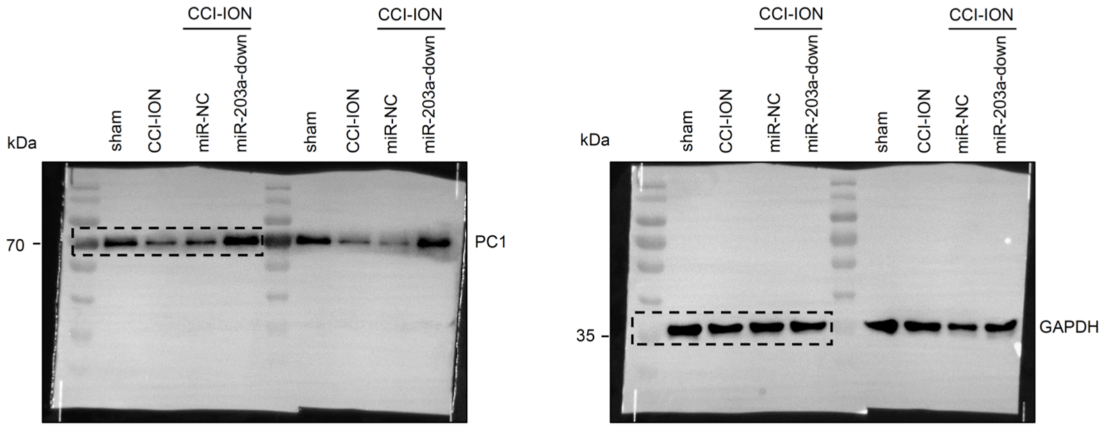

Fig. S21: The decreased expression level of PC1 induced by CCI-ION was reversed by intra-ARC injection of miR-203a-down. Shown are the expanded images of Western blots for PC1 against a loading control, GAPDH, presented in Fig. 6l. Blots are representative of at least three experiments.

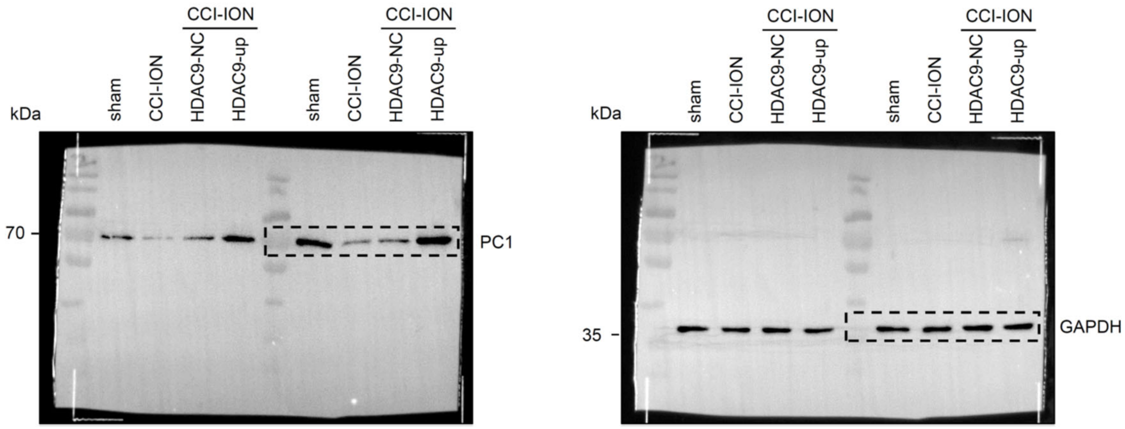

Fig. S22: The decreased expression level of PC1 induced by CCI-ION was reversed by intra-ARC injection of HDAC9-up. Shown are the expanded images of Western blots for PC1 against a loading control, GAPDH, presented in Fig. 6m. Blots are representative of at least three experiments.

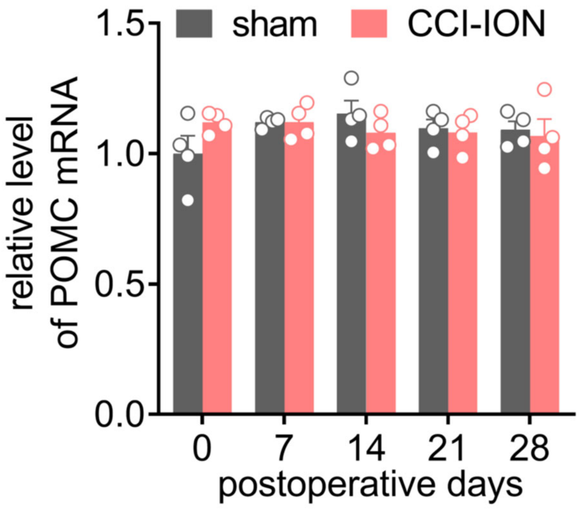

Fig. S23: qPCR analysis of POMC mRNA expression in the ARC following sham or CCI-ION operation. $n = 8$ rats per time point per group.

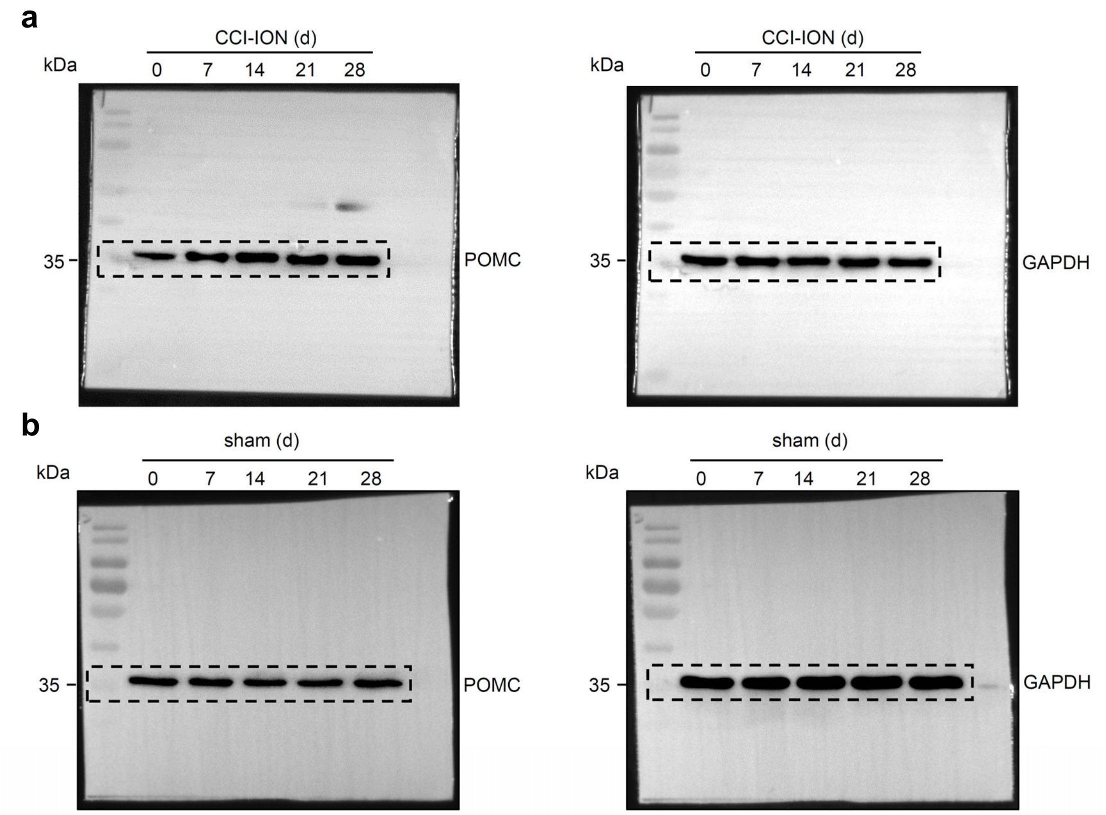

Fig. S24: Protein expression levels of POMC in the ARCs on days 0, 7, 14, 21, and 28 after CCI-ION-operation (*a*) or sham surgery (*b*). Shown are the expanded images of Western blots for POMC against a loading control, GAPDH, presented in Fig. 7b. Blots are representative of at least three experiments.

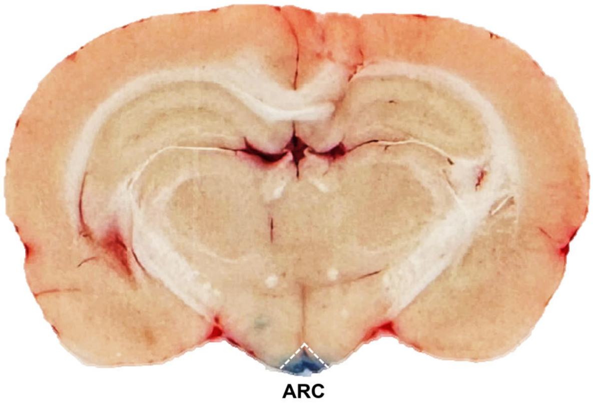

Fig. S25: The injection sites within the ARC were histologically verified by injecting a blue dye. Blue color appears only within the ARC area (outlined by white dashed line), and almost no spreading of the injected reagents into the neighboring brain regions.

Table S1: Primers of miRNAs used in the current study.

Name	RT	Forward	Reverse
miR-203a-3p	GTCGTATCCAGTGCAGGGTCCGAGGTATTCGCACTGGATACGACCTAGTG	CACCGTGGTGAAATGTTTAGGA	
miR-27a-3p	GTCGTATCCAGTGCAGGGTCCGAGGTATTCGCACTGGATACGACGCGGAA	AATCGGCGTTCACAGTGGCTAA	
miR-30a-3p	GTCGTATCCAGTGCAGGGTCCGAGGTATTCGCACTGGATACGACGCTGCA	GCTCGTCCTTTCAGTCGGATGT	
miR-30d-5p	GTCGTATCCAGTGCAGGGTCCGAGGTATTCGCACTGGATACGACCTTCCA	AACGGCTGTAAACATCCCCG	
miR-30e-3p	GTCGTATCCAGTGCAGGGTCCGAGGTATTCGCACTGGATACGACGCTGTA	GCTCGTCCTTTCAGTCGGATGT	
miR-100-5p	GTCGTATCCAGTGCAGGGTCCGAGGTATTCGCACTGGATACGACCACAAG	AACACGTGAACCCGTAGATCCG	
miR-1298	GTCGTATCCAGTGCAGGGTCCGAGGTATTCGCACTGGATACGACTACATC	ACGCCGTTCATTCGGCTGTC	
miR-145-5p	GTCGTATCCAGTGCAGGGTCCGAGGTATTCGCACTGGATACGACAGGGAT	AAGCGACCGTCCAGTTTTCCC	
miR-152-3p	GTCGTATCCAGTGCAGGGTCCGAGGTATTCGCACTGGATACGACCCAAGT	GAGCGCGTCAGTGCATGACA	ATCCAGTGCAGGGTCCGAGG
miR-184	GTCGTATCCAGTGCAGGGTCCGAGGTATTCGCACTGGATACGACACCCTT	AACCGGTGGACGGAGAACTGA	
miR-192-5p	GTCGTATCCAGTGCAGGGTCCGAGGTATTCGCACTGGATACGACGGCTGT	CGCCGCTGACCTATGAATTG	
miR-199a-5p	GTCGTATCCAGTGCAGGGTCCGAGGTATTCGCACTGGATACGACGAACAG	ACGACGCCCAGTGTTCAGA	
miR-322-3p	GTCGTATCCAGTGCAGGGTCCGAGGTATTCGCACTGGATACGACTGTTGC	GCCGAGCAAACATGAAGCGC	
miR-351-5p	GTCGTATCCAGTGCAGGGTCCGAGGTATTCGCACTGGATACGACTCAGGC	AACAGATCCCTGAGGAGCCCTT	
miR-423-3p	GTCGTATCCAGTGCAGGGTCCGAGGTATTCGCACTGGATACGACACTGAG	AAGCTTAAGCTCGGTCTGAGGC	
miR-451-5p	GTCGTATCCAGTGCAGGGTCCGAGGTATTCGCACTGGATACGACAACTCA	CTGCGCGAAAACCGTTACCATT	
U6	GTCGTATCCAGTGCAGGGTCCGAGGTATTCGCACTGGATACGACAAAATA	AGAGAAGATTAGCATGGCCCCTG	

Table S2: Sequences of nucleotides used in the current study.

Name	Forward	Reverse
GAPDH	GTGCTGAGTATGTCGTGGAGT	CAGTCTTCTGAGTGGCAGTGAT
HDAC9	AGCGAGTGTTTGAGGTGG	TTGTTTGGTGAACTGGGAC
POMC	TCACCACGGAAAGCAACCTG	GCTGTTCATCTCCGTTGCCT
EP300	CCTAAATGCTTGCGGACTGC	GCCCAGATATGCCCTGTTGT
CBP	ACTGAAAATCCAGACATTTGGGC	ATCAATCTGCCCTTCCATGCT
GCN5	GGCACAACTCGCTCAGGTTA	CTGACTGATCCTTCCGCCTC
KAT6A	GCCAGAAAGTCCTCGGTCAA	ATTGTGGTGTTTCCGCTTGC
SIRT2	CCAGGGTCCCCAACTCAATC	CTTGCCCTAGGTGGTTGGAG
SIRT7	GTATATTGAAGTCTGCACCTCCT	GAAGCTGAGTCCCACACTTG
PCSK1	TGACTGCATCTCTGCCCAAG	TTCCACTCCAAGCCATCGTC
NR4A2	CAGAGAGACACGGGCTCAAG	GGTCGGTTCAATCCCCCATT
SP1	CCCAGGAAACCCTCCAGAAC	GCATAGGGGCCAAGGTGATT
NFATC2	CAGACTTACCTGGATGACGTT	GAAGGGGTCCAAAGTAGAAGG
SOX10	TGAAGGGCAGAAAAGGGGGA	TCTCAGCCCTGGATGTAGTGA
NFKB1	CCACTCTGGCGCAGAAGTTA	GGAGCTCATCTCATAGTTGTCC A
HADHB	TCCTTCGCAGACTCTAAGATTTC	GGGGCAGACTGTACTTGTGA
MORF4L2	CGCTACGCTTGGAGATTAGG	CCCTGTTTTTATTCCAACCACCT G
ZBTB20	GCGCTGGGGAAGAAGATAAATG	GCAGTCTGTGCGGCTATGA
CAB39	ACAGCTCATTGACTTTGAGGTAT G	TTTGCGAGTGGTTCGTGTCT
PRICKLE2	CTCGGTGAGTGTCCTCCAC	CAGTCCGGTCAACTGGGG
LNX2	CGGGTAGCGTCCTCGTTAG	GGGTGTGTGTCTGCCTGA
COPS7B	GGAGTCTGCAAATCATGCCTCA	CCGCCTACAAAATGTTCCCC
RBM25	TTTTCTTAGCGGCGGACTGG	TGGTGGAGGAAAGCCAGGAAA
ChIP	TTCGTCACGCCCTCTGTTTT	TATGGGCCCCTATGGCTCTC
F1	CCGCTCGAGGTCAGCAGTTGGG CAGGT	CCCAAGCTTAGGAGGGCCATAG GGTTC
F2		CCCAAGCTTGCAGAAAACTGG GCACTAC
F3		CCCAAGCTTAATCCTTCCCGCA AAACT
F4		CCCAAGCTTGCGGACTCGCTTC AAAGA
F5		CCCAAGCTTCTGCACACAGAAG CTCT

Table S3: Agomir, antagomir, siRNAs, and probes used in the current study.

Name	Sense	Antisense
rno-miR-203a-3p agomir	GUGAAAUGUUUAGGACCACUAG	AGUGGUCCUAAACAUUUCACUU
agomir-NC	UUCUCCGAACGUGUCACGUTT	ACGUGACACGUUCGGAGAATT
rno-miR-203a-3p antagomir	CUAGUGGUCCUAAACAUUUCAC	
antagomir-NC	CAGUACUUUUGUGUAGUACAA	
rno-miR-203a-3p mimics	GUGAAAUGUUUAGGACCACUAG	AGUGGUCCUAAACAUUUCACUU
mimics NC	UUCUCCGAACGUGUCACGUTT	ACGUGACACGUUCGGAGAATT
siRNA-HDAC9	CCAUUGAAAUCCAAAGCAATT	UUGCUUUGGAUUUCAAUGGTT
siRNA-PCSK1	CCUGGAACAUGUGCAAUUUTT	AAAUUGCACAUGUUCCAGGTT
siRNA-POMC	CCUACUCCAUGGAGCACUUTT	AAGUGCUCCAUGGAGUAGGTT
siRNA-NC	GGCUCUAGAAAAGCCUAUGCTT	GCAUAGGCUUUUCUAGAGCCTT
rno-miR-203a-3p FISH probe	CTAGTGGTCCTAAACATTTCAC	

Table S4. Information of clinical CSF samples

Group	Sample number	Sex	Age	The injured branch of the trigeminal nerve
control CSF	1#	Female	46	/
	2#	Female	40	/
	3#	Male	55	/
	4#	Male	80	/
	5#	Female	40	/
	6#	Female	53	/
	7#	Male	68	/
Trigeminal neuralgia (TN) Patients CSF	1#	Male	73	Right / V3
	2#	Female	89	Right / V2 V3
	3#	Male	51	Left / V2 V3
	4#	Female	55	Right / V2
	5#	Male	58	Right / V2
	6#	Female	76	Right / V2
	7#	Female	67	Right / V2 V3
	8#	Male	75	Left / V1 V2 V3
	9#	Male	57	Right / V2

Group	number	Male: Female	Age	Distribution by injured branch
control	7	3: 4	54.4 ± 5.5	/
Trigeminal neuralgia	9	5: 4	66.8 ± 4.2	Right / V2: 4<br>Right / V3: 1<br>Right / V2 V3: 2<br>Left / V2 V3: 1<br>Left / V1 V2 V3: 1
