# Supplementary material for: Epigenetic regulation of beta-endorphin synthesis in hypothalamic arcuate nucleus neurons modulates neuropathic pain in a rodent pain model
Source: Nat Commun. 2023 Nov 9;14:7234. doi: 10.1038/s41467-023-43022-7 (PMC10636187; doi:10.1038/s41467-023-43022-7)
Supplement: Supplementary file 3 — Reporting Summary [file 41467_2023_43022_MOESM3_ESM.pdf]

nature portfolio

Corresponding author(s): Jin Tao

Last updated by author(s): Oct 17, 2023

Reporting Summary

Nature Portfolio wishes to improve the reproducibility of the work that we publish. This form provides structure for consistency and transparency in reporting. For further information on Nature Portfolio policies, see our Editorial Policies and the Editorial Policy Checklist.

Statistics

For all statistical analyses, confirm that the following items are present in the figure legend, table legend, main text, or Methods section.

n/a	Confirmed	
☐	☒	The exact sample size (*n*) for each experimental group/condition, given as a discrete number and unit of measurement
☐	☒	A statement on whether measurements were taken from distinct samples or whether the same sample was measured repeatedly
☐	☒	The statistical test(s) used AND whether they are one- or two-sided<br>*Only common tests should be described solely by name; describe more complex techniques in the Methods section.*
☐	☒	A description of all covariates tested
☐	☒	A description of any assumptions or corrections, such as tests of normality and adjustment for multiple comparisons
☐	☒	A full description of the statistical parameters including central tendency (e.g. means) or other basic estimates (e.g. regression coefficient) AND variation (e.g. standard deviation) or associated estimates of uncertainty (e.g. confidence intervals)
☐	☒	For null hypothesis testing, the test statistic (e.g. *F*, *t*, *r*) with confidence intervals, effect sizes, degrees of freedom and *P* value noted<br>*Give P values as exact values whenever suitable.*
☒	☐	For Bayesian analysis, information on the choice of priors and Markov chain Monte Carlo settings
☒	☐	For hierarchical and complex designs, identification of the appropriate level for tests and full reporting of outcomes
☒	☐	Estimates of effect sizes (e.g. Cohen's *d*, Pearson's *r*), indicating how they were calculated

Our web collection on statistics for biologists contains articles on many of the points above.

Software and code

Policy information about availability of computer code

Data collection	Light-Cycler 96 system (Roche)<br>ChemiDoc XRS system (Bio-Rad Laboratories)<br>NIS-Elements F (Nikon)<br>TD-20/20 Luminometer (Turner BioSystems)
Data analysis	GraphPad Prism v8<br>ImageJ v1.52<br>Microsoft Excel 2019

For manuscripts utilizing custom algorithms or software that are central to the research but not yet described in published literature, software must be made available to editors and reviewers. We strongly encourage code deposition in a community repository (e.g. GitHub). See the Nature Portfolio guidelines for submitting code & software for further information.

Data

Policy information about availability of data

All manuscripts must include a data availability statement. This statement should provide the following information, where applicable:

- Accession codes, unique identifiers, or web links for publicly available datasets
- A description of any restrictions on data availability
- For clinical datasets or third party data, please ensure that the statement adheres to our policy

The raw sequencing data have been deposited in the National Center for Biotechnology Information Gene Expression Omnibus (GEO) under the accession number GSE216965 (https://www.ncbi.nlm.nih.gov/geo/query/acc.cgi?acc=GSE216965). The potential targets of miR-203a-3p was obtained from public databases, including TargetScan (www.targetscan.org/), miRanda (www.microrna.org/microrna), miRWalk (mirwalk.umm.uni-heidelberg.de/) and miRDB (www.mirdb.org/). The potential transcription factors involved in the transcriptional regulation of miR-203a-3p was obtained from the public database JASPAR (https://jaspar.genereg.net/). The CpG island in the miR-203a-3p gene promoter region was obtained from the public database MethPrimer (www.urogene.org/cgi-bin/methprimer). All other study data are included in the article and SI Appendix. Source data are provided with this paper. All data that may support the findings of this study are available from the corresponding author upon reasonable request.

Research involving human participants, their data, or biological material

Policy information about studies with human participants or human data. See also policy information about sex, gender (identity/presentation), and sexual orientation and race, ethnicity and racism.

Reporting on sex and gender	Participant sex is included in the Supporting Information (Table S4).
Reporting on race, ethnicity, or other socially relevant groupings	All subjects were of Han Chinese origin.
Population characteristics	Population characteristics are included in the Supporting Information (Table S4).
Recruitment	All patients gave written informed consent to sample collection and data analysis prior to study entry. All CSF samples were collected through the First Affiliated Hospital of Soochow University, and obtained with informed consent compliance with the Ethical Committee of the First Affiliated Hospital of Soochow University (Ethical approval: No.2023-095). A total of 7 healthy subjects (three males and four females) and 9 patients with trigeminal neuralgia (five males and four females) were recruited for this study, and they had not suffered from any other neurological disorder. The reference standard clinical diagnosis for trigeminal neuralgia could be based on defined criteria or judgment of 1 or more experienced doctors (neurologist, pain specialist).
Ethics oversight	Ethical approval was obtained from the the institutional review board of the First Affiliated Hospital of Soochow University Ethics Committee (No.2023-095).

Note that full information on the approval of the study protocol must also be provided in the manuscript.

Field-specific reporting

Please select the one below that is the best fit for your research. If you are not sure, read the appropriate sections before making your selection.

☒ Life sciences ☐ Behavioural & social sciences ☐ Ecological, evolutionary & environmental sciences

For a reference copy of the document with all sections, see nature.com/documents/nr-reporting-summary-flat.pdf

Life sciences study design

All studies must disclose on these points even when the disclosure is negative.

Sample size	No statistical method was used to predetermine sample size. Required experimental sample sizes were estimated based on previous established protocols in the field. The sample sizes were adequate as the differences between experimental groups were reproducible. All n values are clearly indicated within the figure legends. We included a minimum of 3 biological replicates for each group/treatment/timepoint/sex.
Data exclusions	No data exclusions were applied.
Replication	All experiments were performed at least three times with similar results. The findings were reliably reproduced.
Randomization	Animals were randomly assigned into different cages at the time of purchase and randomly assigned into different treatment groups before experiments. No bias in sample allocation was involved.
Blinding	In all of behavior tests, the investigators were blinded to the drug administrations and the groups assignments. All of the drugs were injected

Blinding

by another person. For all other experiments, blinding was not conducted during experiments because data reported for cell and rat experiments were not subjective but rather based on quantitative analyses, which is not influenced by investigator's bias.

Reporting for specific materials, systems and methods

We require information from authors about some types of materials, experimental systems and methods used in many studies. Here, indicate whether each material, system or method listed is relevant to your study. If you are not sure if a list item applies to your research, read the appropriate section before selecting a response.

Materials & experimental systems

n/a	Involved in the study
☐	☒ Antibodies
☐	☒ Eukaryotic cell lines
☒	☐ Palaeontology and archaeology
☐	☒ Animals and other organisms
☒	☐ Clinical data
☒	☐ Dual use research of concern
☒	☐ Plants

Methods

n/a	Involved in the study
☒	☐ ChIP-seq
☒	☐ Flow cytometry
☒	☐ MRI-based neuroimaging

Antibodies

Antibodies used

Primary antibodies for western blot:
anti-H3K9ac (polyclonal, rabbit, 1:1000, Merck Millipore, Cat# 06-942),
anti-H3K14ac (polyclonal, rabbit, 1:1000, Merck Millipore, Cat# 07-353),
anti-H3K18ac (monoclonal, rabbit, 1:1000, Abcam, Cat# ab40888),
anti-H3K27ac (polyclonal, rabbit, 1:1000, Merck Millipore, Cat# 07-360),
anti-H4ac (monoclonal, rabbit, 1:800, Abcam, Cat# ab177790),
anti-H3 (polyclonal, rabbit, 1:100, Abcam, Cat# ab1791),
anti-HDAC9 (monoclonal, rabbit, 1:1000, Abcam, Cat# ab109446),
anti-NR4A2 (monoclonal, mouse, 1:1000, Abcam, Cat# ab41917),
anti-POMC (monoclonal, rabbit, 1:2000, Abcam, Cat# ab210605),
anti-PC1 (monoclonal, rabbit, 1:1000, Abcam, Cat# ab220363),
anti-PC2 (polyclonal, rabbit, 1:1000, Abcam, Cat# ab3533),
anti-glyceraldehyde 3-phosphate dehydrogenase (GAPDH, Monoclonal, mouse, 1:1000, Cell Signaling Technology, Cat# 97166).

Secondary antibodies for western blot:
goat anti-rabbit horseradish peroxidase secondary antibody (1:5000, R&D Systems, Cat# HAF008),
goat anti-mouse horseradish peroxidase secondary antibody (1:5000, R&D Systems, Cat# HAF007).

Primary antibodies for Immunofluorescent staining:
c-Fos (monoclonal, mouse, 1:300, Abcam, Cat# ab208942),
HDAC9 (monoclonal, rabbit, 1:300, Abcam, Cat# ab109446),
H3K18ac (monoclonal, rabbit, 1:300, Abcam, Cat# ab40888),
NR4A2 (monoclonal, mouse, 1:300, Abcam, Cat# ab41917),
PC1 (monoclonal, rabbit, 1:300, Abcam, Cat# ab220363),
NeuN (monoclonal, mouse, 1: 300, Merck Millipore, Cat# MAB377),
GFAP (monoclonal, mouse, 1:300, Cell Signaling Technology, Cat# 3670),
CD11b/c (monoclonal, mouse, 1:200, Abcam, Cat# ab1211),
β-endorphin (monoclonal, mouse, 1:200, Abcam, Cat# ab54205).

Secondary antibodies for Immunofluorescent staining:
Alexa Fluor® 555-conjugated goat anti-rabbit IgG (1:300, Cell Signaling Technology, Cat# 4413S),
Alexa Fluor® 488-conjugated goat anti-rabbit IgG (1:300, Cell Signaling Technology, Cat# 4412S),
Alexa Fluor® 488-conjugated goat anti-mouse IgG (1:300, Cell Signaling Technology, Cat# 4408S).

Validation

All antibodies for western blot and Immunofluorescent staining were well-recognized clones in the field and validated by the manufacturers. These antibodies are further validated and routinely used in our lab.

Primary antibodies for western blot:
anti-H3K9ac (https://www.sigmaaldrich.cn/CN/zh/product/mm/06-942),
anti-H3K14ac (https://www.sigmaaldrich.cn/CN/zh/product/mm/07353),
anti-H3K18ac (https://www.abcam.cn/products/primary-antibodies/histone-h3-acetyl-k18-antibody-ep959y-chip-grade-ab40888.html),
anti-H3K27ac (https://www.sigmaaldrich.cn/CN/zh/product/mm/07-360),
anti-H4ac (https://www.abcam.cn/products/primary-antibodies/histone-h4-acetyl-k5--k8--k12--k16-antibody-epr16606-ab177790.html),
anti-H3 https://www.abcam.cn/products/primary-antibodies/histone-h3-antibody-nuclear-marker-and-chip-grade-ab1791.html),
anti-HDAC9 (https://www.abcam.cn/products/primary-antibodies/hdac9-antibody-epr5223-ab109446.html),

anti-NR4A2 (https://www.abcam.cn/products/primary-antibodies/nurr1-antibody-n1404-ab41917.html),
anti-POMC (https://www.abcam.cn/products/primary-antibodies/pomc-antibody-epr17571-ab210605.html88-3),
anti-PC1 (https://www.abcam.cn/products/primary-antibodies/pc13-antibody-epr21908-ab220363.html),
anti-PC2 https://www.abcam.cn/products/primary-antibodies/pcsk2-antibody-ab3533.html ab3533),
anti-glyceraldehyde 3-phosphate dehydrogenase (https://www.cellsignal.cn/products/primary-antibodies/gapdh-d4c6r-mouse-mab/97166).

Secondary antibodies for western blot:
goat anti-rabbit horseradish peroxidase secondary antibody (https://www.rndsystems.com/cn/products/rabbit-igg-horseradish-peroxidase-conjugated-antibody_haf008),
goat anti-mouse horseradish peroxidase secondary antibody (https://www.rndsystems.com/cn/products/mouse-igg-horseradish-peroxidase-conjugated-antibody_haf007).

Primary antibodies for Immunofluorescent staining:
c-Fos (https://www.abcam.cn/products/primary-antibodies/c-fos-antibody-2h2-ab208942.html),
HDAC9 (https://www.abcam.cn/products/primary-antibodies/hdac9-antibody-epr5223-ab109446.html),
H3K18ac (https://www.abcam.cn/products/primary-antibodies/histone-h3-acetyl-k18-antibody-ep959y-chip-grade-ab40888.html),
NR4A2 (https://www.abcam.cn/products/primary-antibodies/nurr1-antibody-n1404-ab41917.html),
PC1 (https://www.abcam.cn/products/primary-antibodies/pc13-antibody-epr21908-ab220363.html),
NeuN (https://www.sigmaaldrich.cn/CN/zh/product/mm/mab377),
GFAP (https://www.cellsignal.cn/products/primary-antibodies/gfap-ga5-mouse-mab/3670),
CD11b/c (https://www.abcam.cn/products/primary-antibodies/cd11b--cd11c-antibody-ox42-ab1211.html08-1),
β-endorphin (https://www.abcam.cn/products/primary-antibodies/beta-endorphin-antibody-b-3115-ab54205.html).

Secondary antibodies for Immunofluorescent staining:
Alexa Fluor® 555-conjugated goat anti-rabbit IgG (https://www.cellsignal.cn/products/secondary-antibodies/anti-rabbit-igg-h-l-f-ab-2-fragment-alexa-fluor-555-conjugate/4413),
Alexa Fluor® 488-conjugated goat anti-rabbit IgG (https://www.cellsignal.cn/products/secondary-antibodies/anti-rabbit-igg-h-l-f-ab-2-fragment-alexa-fluor-488-conjugate/4412),
Alexa Fluor® 488-conjugated goat anti-mouse IgG (https://www.cellsignal.cn/products/secondary-antibodies/anti-mouse-igg-h-l-f-ab-2-fragment-alexa-fluor-488-conjugate/4408).

Eukaryotic cell lines

Policy information about cell lines and Sex and Gender in Research

Cell line source(s)	PC12 cells and HEK293 cells were purchased from Cell Bank of Type Culture Collection of the Chinese Academy of Sciences (Shanghai Institute of Cell Biology).
Authentication	PC12 cells and HEK293 cells were purchased from Cell Bank of Type Culture Collection of the Chinese Academy of Sciences (Shanghai Institute of Cell Biology). No further authentication procedures were performed.<br>HEK293 https://www.cellbank.org.cn/search-detail.php?id=910<br>PC12 https://www.cellbank.org.cn/search-detail.php?id=548
Mycoplasma contamination	Cells were not mycoplasma positive.
Commonly misidentified lines (See ICLAC register)	No commonly misidentified cell lines were used in this study.

Animals and other research organisms

Policy information about studies involving animals; ARRIVE guidelines recommended for reporting animal research, and Sex and Gender in Research

Laboratory animals	Animals were purchased from Shanghai SLAC Laboratory Animal Co., Ltd. (Shanghai, China) and maintained at the Soochow University Animal Facility. Adult Sprague–Dawley rats (male and female, 8 – 10 weeks) were housed (three rats per cage with soft bedding) in specific temperature- and humidity-controlled facilities on a 12/12-h light-dark cycle with food and water available ad libitum. Every effort was made to minimize suffering and the number of animals used.
Wild animals	No wild animals were used in this study.
Reporting on sex	Both male and female rats were incorporated into our experimental design. To avoid any influence of the menstrual cycle on pain-related behaviors, rats used in the study referred to the male ones unless otherwise specified. Key experiments were conducted with female rats, yielding data that aligns closely with the findings we had obtained from male rats.
Field-collected samples	No field-collected samples were used in this study.
Ethics oversight	All animal procedures were approved by the Animal Care and Use Committee of Soochow University strictly in accordance with the National Institutes of Health (NIH) guidelines for animal research and the International Association for the Study of Pain.

Note that full information on the approval of the study protocol must also be provided in the manuscript.
